# MRI in the Diagnosis of Bucket Handle Tears: What Is the Current Situation?

**DOI:** 10.7759/cureus.43324

**Published:** 2023-08-11

**Authors:** Emre Pakdemirli, Turay Cesur, İbrahim Bozkurt

**Affiliations:** 1 Radiology, Ankara Bilkent City Hospital, Ankara, TUR; 2 Orthopaedics and Traumatology, Ankara Bilkent City Hospital, Ankara, TUR

**Keywords:** signs, meniscus tear, mri, meniscal tear, bucket handle tear

## Abstract

Introduction

The purpose of this study was to determine the utility of current magnetic resonance imaging (MRI) in the diagnosis of bucket-handle meniscal tears.

Materials and methods

Patients treated for arthroscopic meniscal tears between March 2019 and March 2022 were reviewed. The current study included all patients with bucket handle tears diagnosed arthroscopically and having MRI scans (n=51). A control group of 58 individuals with similar demographic characteristics and meniscal tears apart from bucket handle tears was also formed. The assessment of bucket handle and non-bucket handle tears was performed blindly by a musculoskeletal (MSK) radiologist with 20 years of experience and a trainee radiologist, achieving consensus on group allocation. The MRIs were examined for various findings, including the presence of a bucket handle tear, tear location, presence of anterior cruciate ligament (ACL) rupture, intercondyler notch sign, double anterior horn sign, flipped meniscus sign, double posterior cruciate ligament (PCL) sign, absent bow sign, and the disproportionate posterior horn sign. These well-known signs, detailed in the literature, were evaluated. Additionally, less studied and less commonly known signs such as the V sign and double anterior cruciate ligament sign were assessed. The V sign appears similarly to the letter V, resulting from the displacement of the bucket handle tear and the angle of the intact meniscus on axial images. The double anterior cruciate ligament sign is the appearance formed by the compression of the displaced meniscal part behind the anterior cruciate ligament in bucket handle tears.

Results

Following the retrospective evaluation of MRI scans, 44 out of 51 tears diagnosed as bucket handle tears by arthroscopy were accurately identified (sensitivity: 86.27%). The same conclusion was reached for MRI scans in 52 out of 58 tears where arthroscopy did not detect a bucket handle tear (specificity: 89.66%). The most prevalent MRI signs in patients with bucket handle tears identified by arthroscopy in the study were the intercondylar notch sign (84.31%), V sign (72.55%), double PCL sign (56.86%), double anterior horn sign (49.02%), absent bow sign (43.14%), flipped meniscus sign (19.61%), disproportionate posterior horn sign (9.80%), and double ACL sign (5.88%). The intercondylar notch sign, V sign, and double PCL sign exhibited the highest sensitivity, while flipped meniscus, disproportionate posterior horn, and double ACL sign demonstrated the highest specificity.

Conclusion

MRI demonstrates a high level of sensitivity and specificity in identifying meniscal bucket handle tears, particularly when considering the eight MRI signs investigated in this study.

## Introduction

A meniscus tear is one of the most prevalent sports-related injuries, and it usually requires surgery due to knee instability and functionality [1]. An attached fragment that displaces away from the meniscus together with a vertical, longitudinal, or oblique tear is known as a "bucket-handle" meniscal tear. The "handle" is frequently formed by the inner meniscal fragment being moved into the intercondylar notch. The incidence of a bucket-handle tear is 10-19% [2,3]. These tears frequently coexist with anterior cruciate ligament (ACL) injuries and are more frequently observed in the medial meniscus as compared to the lateral meniscus [4,5].

Whenever possible, meniscal repair has become the treatment of choice for meniscal tears [6]. This is especially the case with bucket-handle meniscal tears (BHMTs), which involve large portions of the meniscus [7,8]. Detection of a displaced meniscal fragment in a bucket-handle tear is crucial because removing or reattaching the fragment requires arthroscopy. MRI is currently the best imaging technique for assessing meniscal abnormalities due to its non-invasive nature and high level of specificity and sensitivity [9,10].

**Figure 1:**
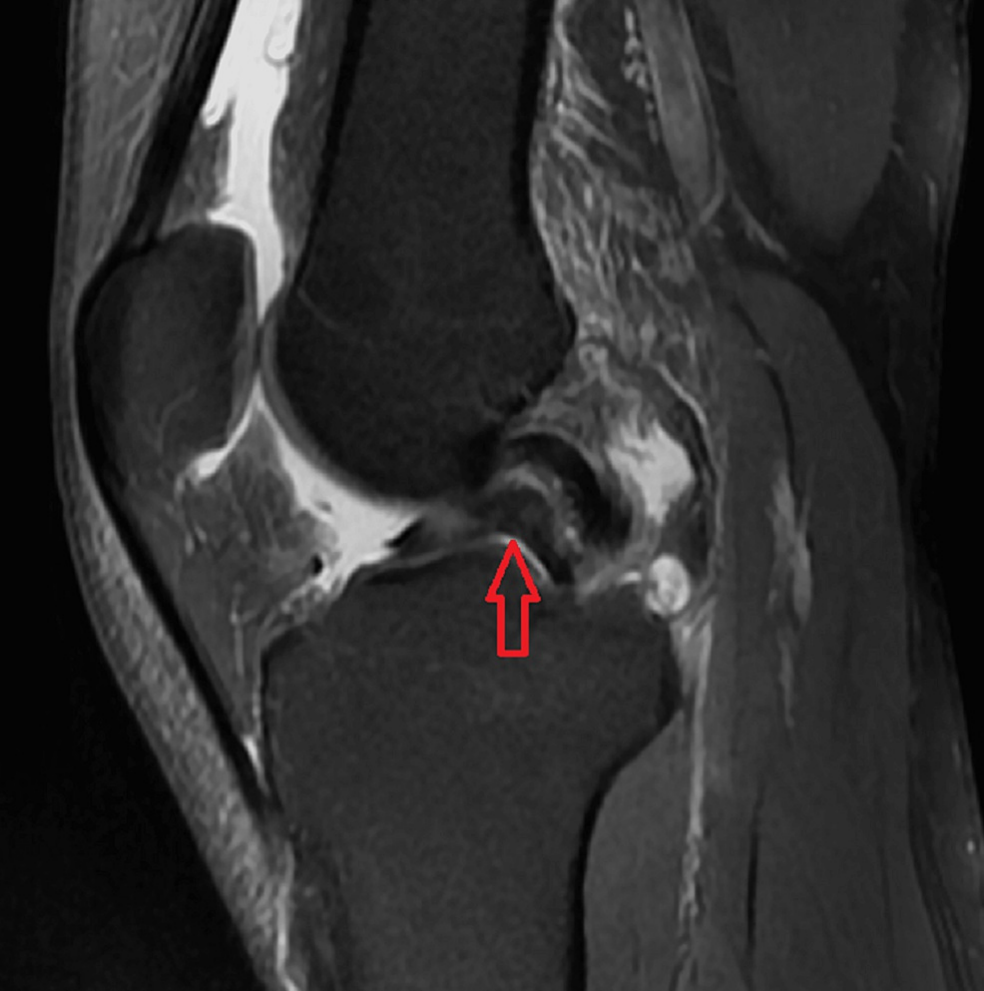
The sagittal image showing the bucket handle tear (red arrow) forming the double posterior cruciate ligament sign.

**Figure 2:**
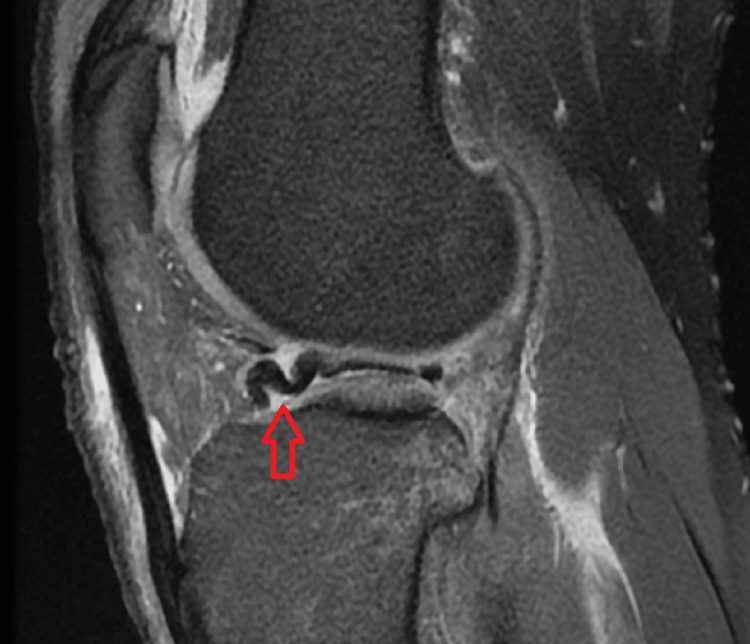
The sagittal image showing the bucket handle tear displaced through the anterior horn forming the flipped meniscus sign (red arrow).

**Figure 3:**
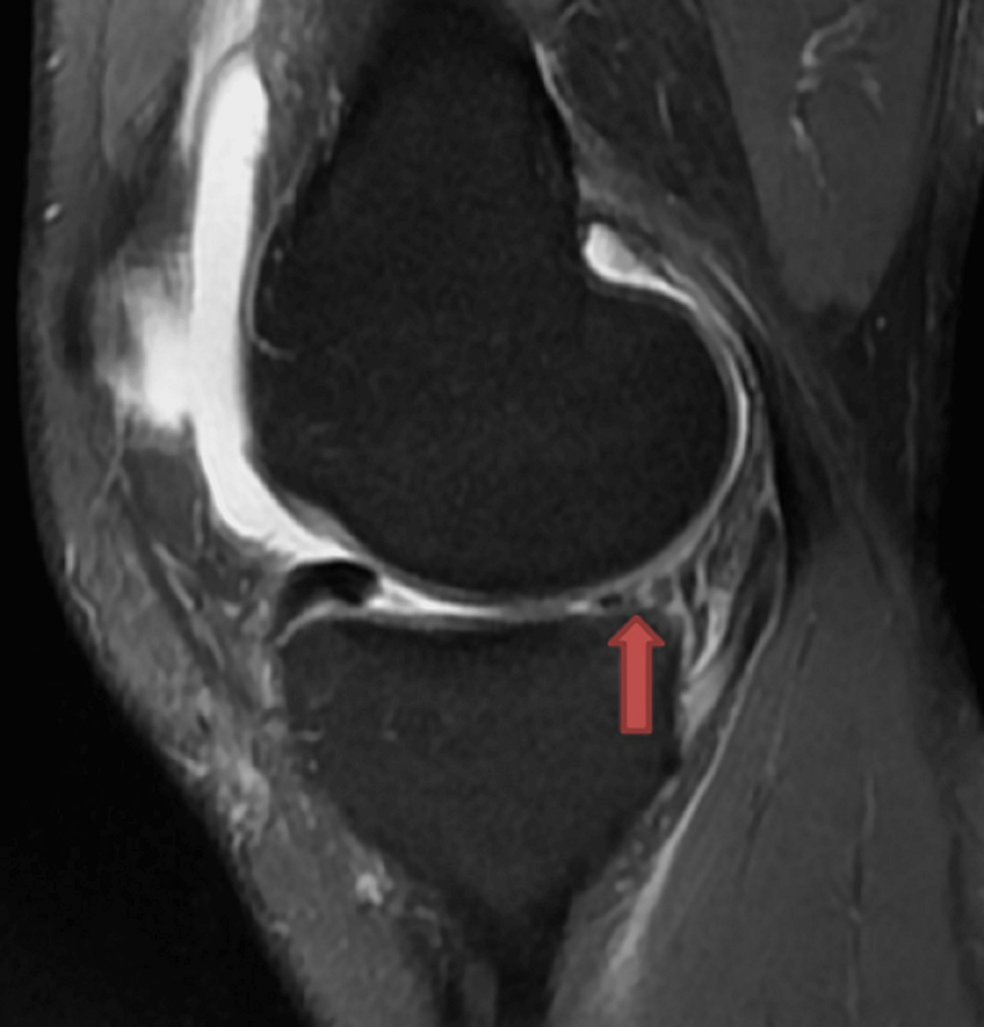
The sagittal image showing the bucket handle tear forming the absent bow tie sign (red arrow).

**Figure 4:**
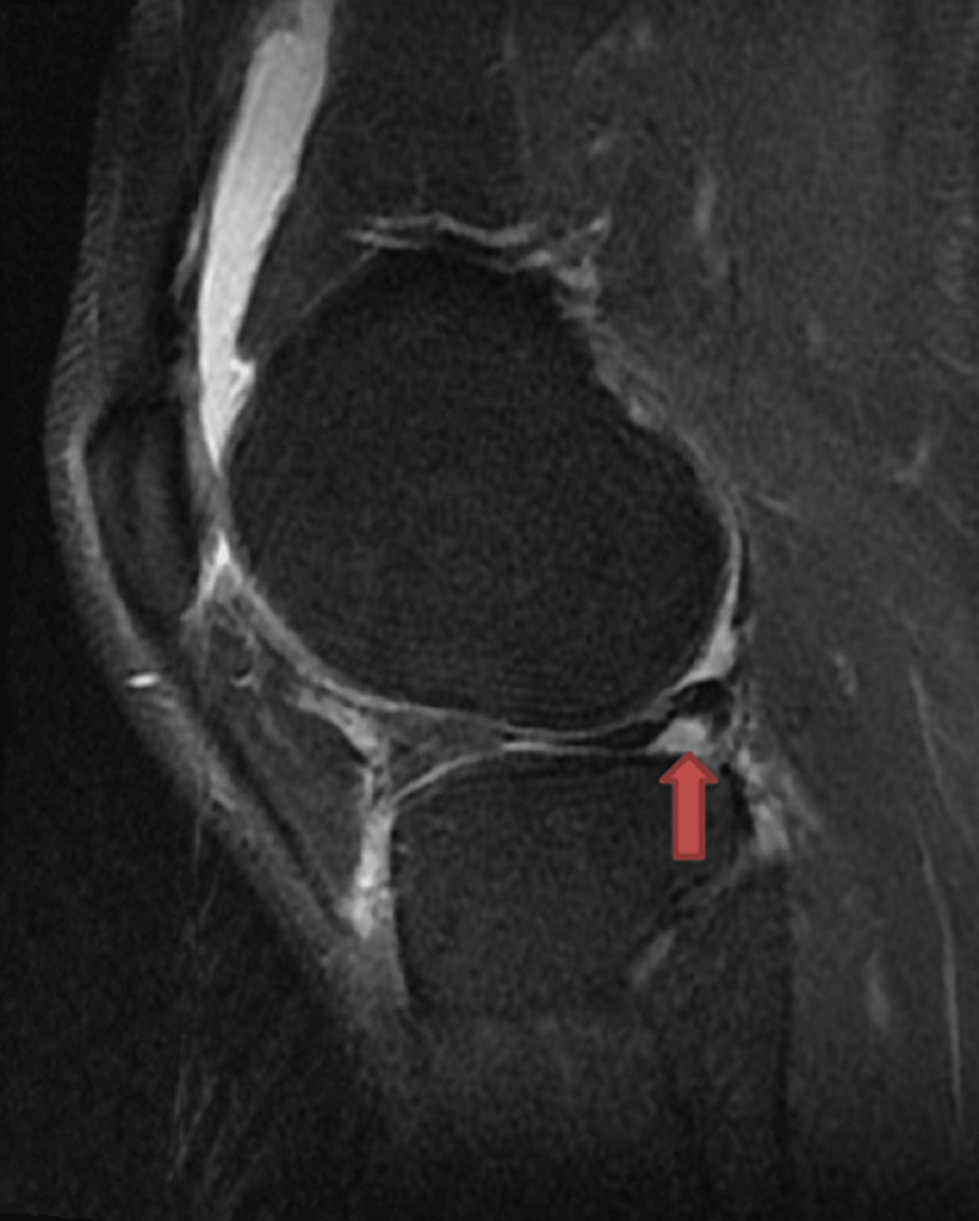
The sagittal image showing the bucket handle tear forming the disproportional posterior horn sign (red arrow).

**Figure 5:**
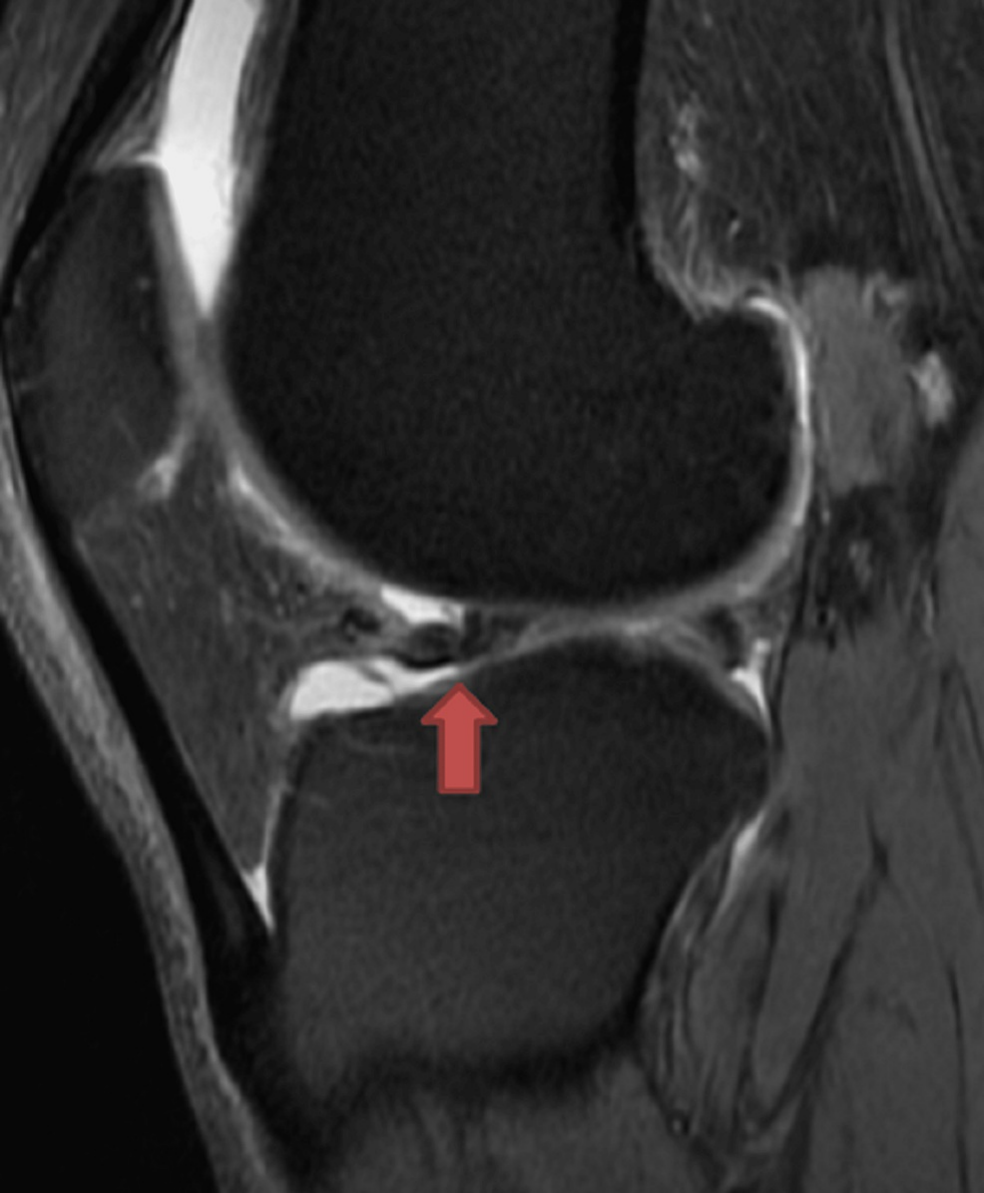
The sagittal image showing the bucket handle tear forming the double anterior horn sign (red arrow).

**Figure 6:**
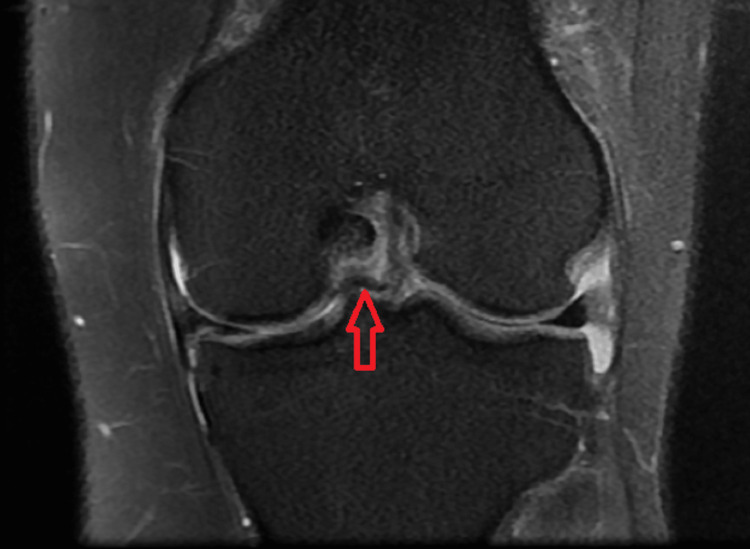
The coronal image showing the bucket handle tear at the intercondylar distance forming the intercondylar notch sign (red arrow).

**Figure 7:**
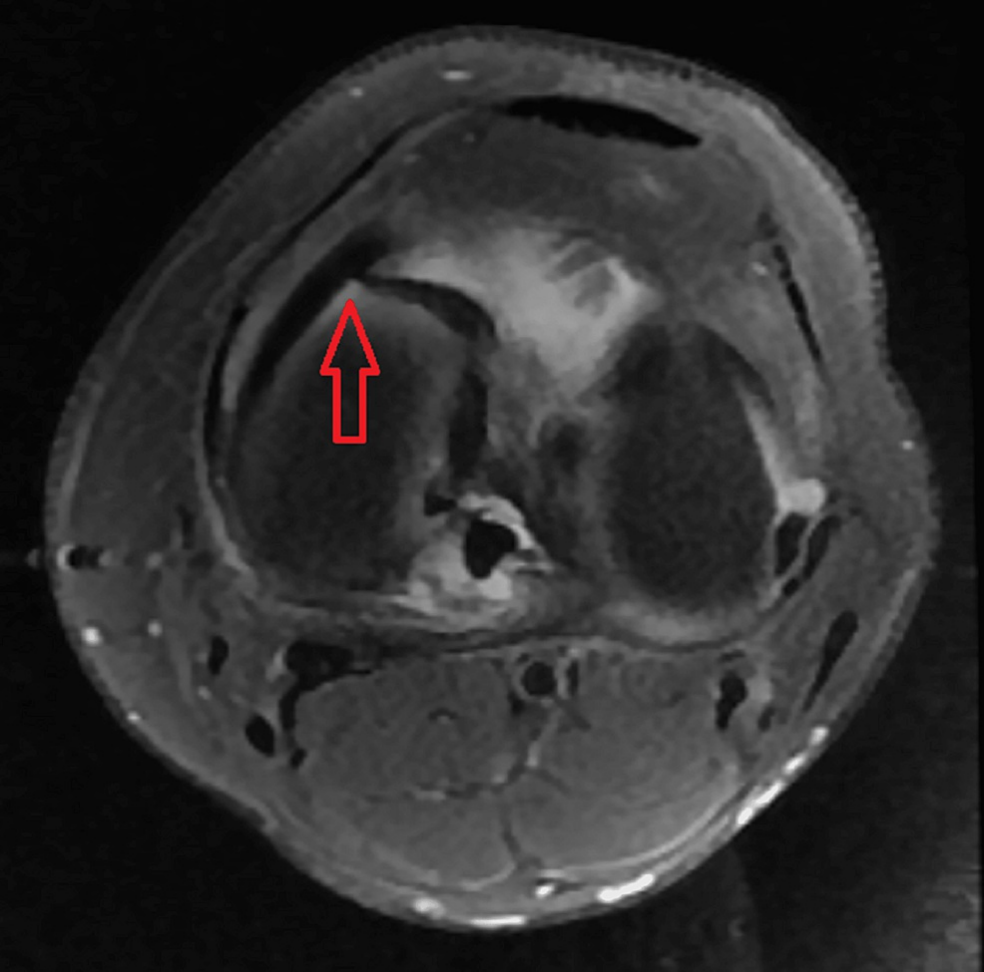
The axial image showing the bucket handle tear forming the V sign (red arrow).

**Figure 8:**
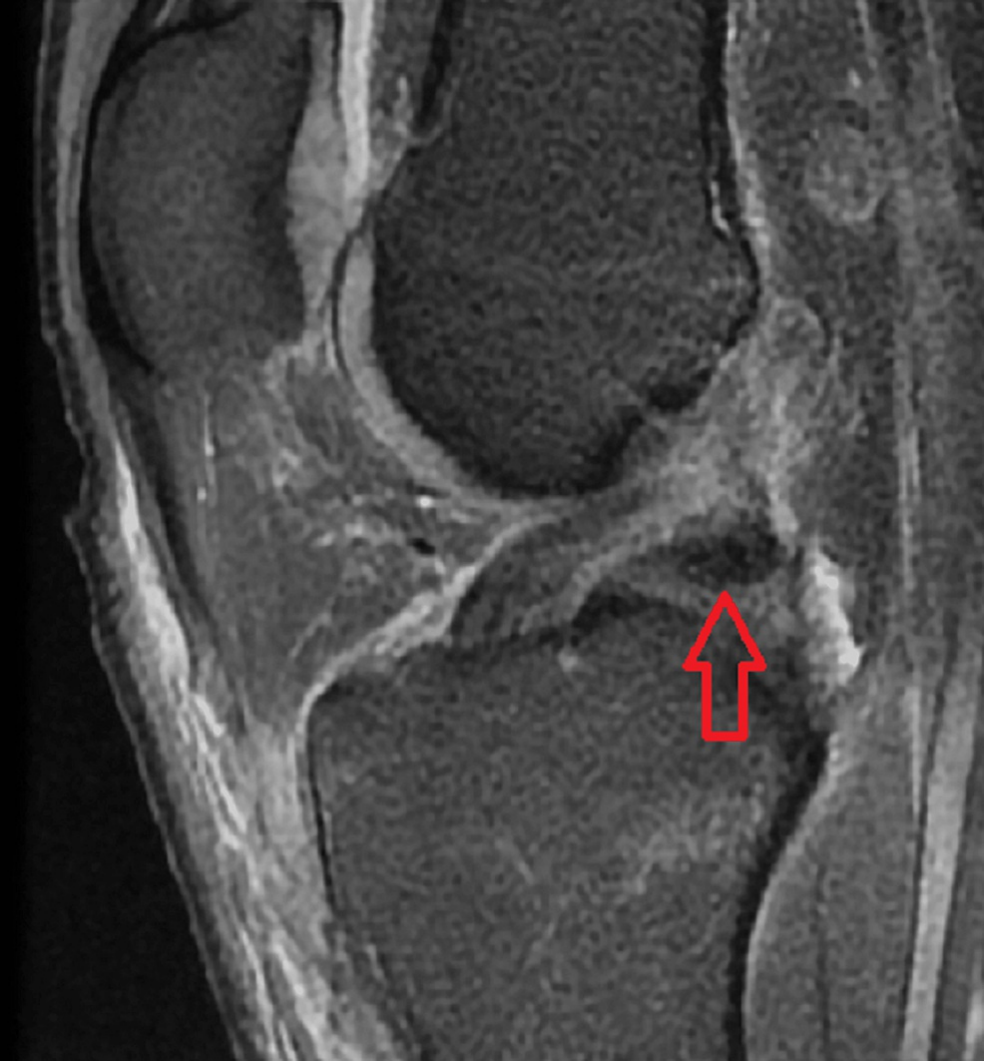
The sagittal image shows the bucket handle tear forming the double anterior cruciate ligament sign (red arrow).

**Figure 9:**
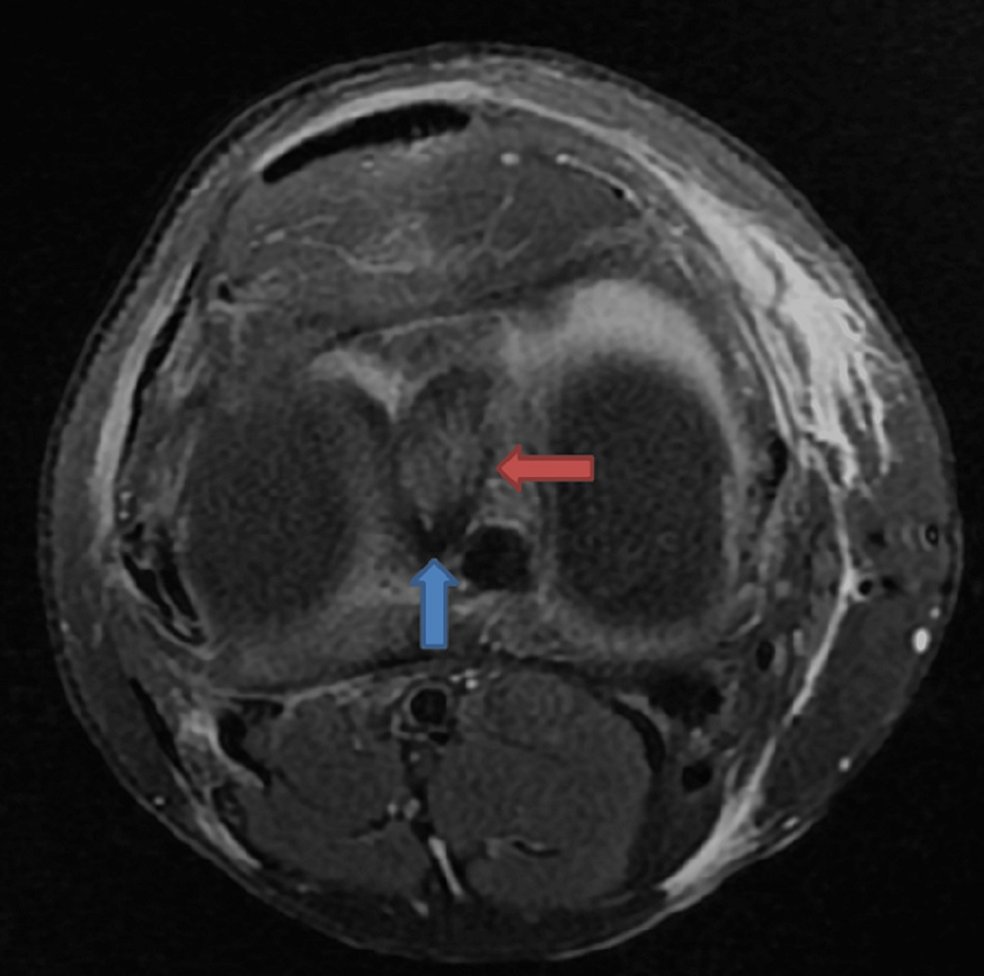
The axial image showing the bucket handle tear (blue arrow) placed posterior to the anterior cruciate ligament (ACL) (red arrow) forming the double ACL sign in sagittal images.

Various MRI signs have been previously discussed in the literature. Sagittal imaging is used to describe a bucket handle tear, such as the double posterior cruciate ligament (PCL) sign [4,11,12] (Figure 1), flipped meniscus sign [4,13] (Figure 2), absent bow-tie sign [14,15] (Figure 3), disproportional posterior horn sign [16,17,18] (Figure 4), and double anterior horn sign [17,18,19] (Figure 5). Coronal imaging reveals the intercondylar notch sign [4,11,17,18] (Figure 6), while axial imaging shows the V-sign [5] (Figure 7), which has been recently described. Most of these signs are well known and extensively discussed in the literature. However, to our knowledge, no study has been published evaluating all of these MR imaging signs of meniscal bucket-handle tears, including the recently identified V-sign [5] and double ACL sign [20,21] (Figure 8,9), in a larger population. This study aims to assess both the recently proposed and well-established MR imaging signs of meniscal bucket-handle tears and to explore any relationships between arthroscopy and MRI findings.

## Materials and methods

The study was initiated in accordance with the ethics committee approval numbered E2-22-2466, granted at the meeting held on September 28, 2022, by the Ethics Committee No. 2 of Ankara Bilkent City Hospital, Ankara, Turkey.

All patients treated for arthroscopic meniscal tears between March 2019 and March 2022 were reviewed. Those diagnosed with bucket handle tears via arthroscopy who also underwent MRI scans were included in the current study (n=51). A control group of 58 individuals with similar demographic characteristics and meniscal tears other than bucket handle tears was established. The assessment of bucket handle and non-bucket handle tears was conducted blindly by an MSK radiologist with 20 years of experience, along with a trainee radiologist reaching a consensus.

The MRIs were examined for the presence of the following findings: the presence of a bucket handle tear, the side and location of the tear (medial or lateral), whether it was accompanied by ACL rupture, and the double PCL sign (Figure 1), flipped meniscus sign (Figure 2), absent bow sign (Figure 3), disproportionate posterior horn sign (Figure 4), double anterior horn sign (Figure 5), and intercondylar notch sign (Figure 6).

In addition, an assessment was conducted regarding the V sign (Figure 7) and the double ACL sign (Figures 8, 9), both of which have received limited attention in the existing literature and are not commonly recognized in routine practice. The V sign presents a similar appearance to the letter "V," arising from the displacement of the bucket handle tear and the angle of the intact meniscus in axial images [5]. The double ACL sign manifests as an appearance formed by the compression of the displaced meniscal portion anteriorly or posteriorly to the ACL in lateral meniscus bucket handle tears [20,21].

Additionally, the amount of intra-articular fluid in the evaluations made with knee MRI was visually graded as absent, mild, moderate, or severe. The MR imaging exams were performed using a 3T General Electric Signa Pioneer. Each examination included sagittal T1w FSE, sagittal fat saturated PD FSE, axial fat saturated PD FSE, coronal fat saturated PDFS, and sagittal T2w 3D cube.

The arthroscopy procedure was performed by orthopedic surgeons in our hospital with 10 years of experience in arthroscopy. The patients were operated on in the supine position under general or spinal anesthesia. The knee joint was visualized from the anterolateral and anteromedial portals using a 30-degree arthroscope. The medial and lateral meniscus, ACL, and PCL were evaluated. Bucket handle meniscal tears were repaired with sutures, and accompanying ligament injuries were reconstructed arthroscopically.

IBM SPSS Statistics for Windows, Version 20 (Released 2017; IBM Corp., Armonk, New York, United States) statistical program was used to evaluate the findings obtained in our study. The normal distribution was evaluated using the Kolmogorov-Smirnov test. Descriptive statistics for numerical variables (minimum, maximum, mean, median, standard deviation) were provided, and numbers and percentages were given for categorical variables. The Pearson chi-square test was used to compare categorical variables in the study, with a p-value <0.05 considered significant.

## Results

Of the 109 patients included in the study, 82 (75.23%) were male and 27 (24.77%) were female. The mean age of the patients was calculated as 38.45±12.78 years. Among the patients included in the study, 51 had bucket handle tears (40 males and 11 females) and 58 (42 males and 16 females) had non-bucket handle meniscal tears arthroscopically. Among the patients diagnosed with bucket handle tears by arthroscopy and subsequently confirmed by MRI scans, 44 out of 51 were accurately identified (sensitivity: 86.27%). Similarly, MRI scans correctly identified the absence of bucket handle tears in 52 out of 58 patients as determined by arthroscopy (specificity: 89.66%) (Table 1).

**Table 1:** Comparison of MRI and arthroscopy in terms of diagnosis of bucket handle tear

		Bucket Handle (Arthroscopy)			
		Not seen		Seen	
		N	%	N	%
Bucket handle (MRI)	Not seen	52	89.66	7	13.73
	Seen	6	10.34	44	86.27

According to the arthroscopy reports, 50.9% (n=26) of the patients exhibited a bucket handle tear in the right knee, while the remaining 49.1% (n=25) had it in the left knee. Through MRI scans, bucket handle tears were diagnosed in 50 out of 109 patients, while 59 were identified with non-bucket handle meniscal tears. MRI-diagnosed bucket handle tears were distributed as 48.00% on the right (n=24) and 52.00% on the left (n=26).

Among the patients with bucket handle tears as determined by arthroscopy, 84.31% (n=43) were located medially, and 15.68% (n=8) were located laterally. In contrast, among those identified with bucket handle tears on MRI, 88% (n=44) were located medially, and 12% (n=6) were located laterally.

The arthroscopy report of one of the 109 patients in the study did not provide information about the ACL. Additionally, the ACL-related arthroscopy result of one patient was considered suspicious. Among those whose arthroscopic report was suitable for the research, 62.04% (n=67) did not have ACL rupture, while 37.04% (n=40) exhibited ACL rupture. The amount of joint fluid was assessed by MRI, and it was classified as mild in 22.94% (n=25), moderate in 38.53% (n=42), and severe in 25.69% (n=28) (Table 2).

**Table 2:** ACL rupture and joint fluid status of all patients ACL: anterior cruciate ligament

		N	%
ACL rupture	Negative	67	62.04
	Positive	40	37.04
	Suspicious	1	0.93
Joint fluid status	Normal	14	12.84
	Low	25	22.94
	Mild	42	38.53
	Severe	28	25.69

The most common MRI findings in patients with bucket handle tear, as determined by arthroscopy and included in the study, were as follows: intercondylar notch sign (84.31%), V sign (72.55%), double PCL sign (56.86%), double anterior horn sign (49.02%), absent bow sign (43.14%), flipped meniscus sign (19.61%), disproportionate posterior horn sign (9.80%), and double ACL sign (1.96%) (Table 3).

**Table 3:** Number and percentage of the MRI signs of the arthroscopically proved bucket handle tears ACL: anterior cruciate ligament, PCL: posterior cruciate ligament

	Negative			Positive	
	N	%	N		%
Intercondyler notch	8	15.69	43		84.31
V sign	14	27.45	37		72.55
Double PCL	22	43.14	29		56.86
Double anterior horn	26	50.98	25		49.02
Absent bow	29	56.86	22		43.14
Flipped meniscus	41	80.39	10		19.61
Disproportionate posterior horn	46	90.20	5		9.80
Double ACL	48	94.12	3		5.88

In the study, the sensitivity and specificity of MR imaging findings in detecting bucket handle tears through arthroscopy were calculated. The highest sensitivity was observed for intercondylar notch, V sign, and double PCL, while the highest specificity was noted for flipped meniscus, disproportionate posterior horn, and double ACL (Table 4).

**Table 4:** Sensitivity and specificity of MRI findings in detecting arthroscopic bucket handle tear ACL: anterior cruciate ligament, PCL: posterior cruciate ligament

MR findings	Sensitivity % (TP/TP+FN)	Specifity % (TN/TN+FP)
Intercondyler notch sign	84.31 (43/51)	93.10 (54/58)
V sign	72.55 (37/51)	91.38 (53/58)
Double PCL sign	56.86 (29/51)	100 (58/58)
Double anterior horn sign	49.02 (25/51)	94.83 (55/58)
Absent bow sign	43.14 (22/51)	94.83 (55/58
Flipped meniscus sign	19.61 (10/51)	98.28 (57/58)
Disproportionate posterior horn sign	9.80 (5/51)	98.28 (57/58)
Double ACL sign	1.96 (1/51)	98.28 (57/58)

Prediagnosis was documented for 97 of the patients included in the study. Among them, meniscopathy was reported in 63.92% (n=62), ACL rupture in 23.71% (n=23), bucket handle tear in 4.12% (n=4), and knee pain in 3.09% (n=3). Other patients were queried about avascular necrosis, VNS, enchondroma, patellar dislocation, and MCL rupture (Table 5).

**Table 5:** Clinical information of the patients ACL: anterior cruciate ligament, MCL: medial collateral ligament, VNS: villonodular synovitis

		N	%
Clinical information	Meniscopathy	62	63.92
	ACL rupture	23	23.71
	Bucket handle tear	4	4.12
	Knee pain	3	3.09
	Avascular necrosis	1	1.03
	VNS	1	1.03
	Encondroma	1	1.03
	Patellar dislocation	1	1.03
	MCL rupture	1	1.03

A bucket handle tear was identified in 43.55% (n=27) of the 62 patients initially diagnosed with meniscopathy based on arthroscopy. ACL rupture was confirmed by arthroscopy in 69.57% (n=16) of the 23 patients suspected to have an ACL rupture. Arthroscopy revealed a bucket handle tear in 75% (n=3) of the 4 patients initially suspected to have such a tear (Table 6).

**Table 6:** Comparison of patients' clinical information and arthroscopy findings ACL: anterior cruciate ligament

Meniscopathy	Bucket Handle Tear (Arthroscopy)							
	Unseen					Seen		
	N			%		N		%
	35			56.45		27		43.55
ACL rupture		ACL Rupture (Arthroscopy)						
		Negative			Positive			
		N	%		N		%	
		7	30.43		16		69.57	
Bucket handle tear		Bucket Handle Tear (Arthroscopy)						
		Unseen			Seen			
		N	%		N		%	
		1	25.00		3		75.00	

ACL rupture was positive in 46.94% (n=23) of the patients who were found to have bucket handle tears by arthroscopy and MR imaging, while it was found to be 29.31% (n=17) in meniscal tears other than bucket handle tears. No statistically significant relationship was found between the groups (p>0.05) (Table 7).

**Table 7:** Comparison of bucket handle tear and ACL rupture in arthroscopy and MRI ^a^Pearson chi-square test ACL: anterior cruciate ligament, MRI: magnetic resonance imaging

		ACL rupture				P
		Unseen		Seen		
		N	%	N	%	
Bucket handle tear (arthroscopy)	Unseen	41	70.69	17	29.31	0.060^a^
	Seen	26	53.06	23	46.94	
Bucket handle tear (MRI)	Negative	41	70.69	17	29.31	0.060^a^
	Seen	26	53.06	23	46.94	

The incidence of detecting substantial joint fluid in patients with a bucket handle tear after arthroscopy and MR imaging was found to be statistically significantly higher than in those without a bucket handle tear (p<0.01) (Table 8). Conversely, the normal detection rate of joint fluid in patients with bucket handle tears on MRI was found to be statistically significantly lower than in those without bucket handle tears (p<0.01) (Table 8).

**Table 8:** Comparison of bucket handle tear and joint fluid amount ^a^Pearson chi-square test *p < 0.01 MRI: magnetic resonance imaging

		Joint Fiuid									P
		Normal		Mild		Moderate		Severe			
		N	%	N	%	N	%	N	%		
Bucket handle tear (arthroscopy)	Unseen	10	17.24	16	27.59	27	46.55	5	8.62	0.000^a,^*	
	Seen	4	7.84	9	17.65	15	29.41	23	45.10		
Bucket handle tear (MRI)	Unseen	11	18.64	16	27.12	27	45.76	5	8.47	0.000^a,^*	
	Seen	3	6.00	9	18.00	15	30.00	23	46.00		

## Discussion

We intended to find out how effectively MRI could identify meniscal bucket handle tears. This study adds to the extensive literature on the use of MRI to detect meniscal bucket handle tears for several reasons. In our study, the number of patients and controls was slightly higher than in other similar studies in the literature. In addition, we believe that we compared the highest number of MRI signs we could reach in the literature on this subject.

We found that MRI exhibited a sensitivity of 86.27% and a specificity of 89.66% in detecting bucket handle tears. These results are consistent with existing literature concerning MRI sensitivity in diagnosing bucket handle tears. A parallel study discovered an overall sensitivity of 93% (28/30) when juxtaposed with arthroscopy [4]. According to Aydıngöz et al., MR imaging demonstrated an overall sensitivity and positive predictive value of 90% for identifying meniscal bucket handle tears [17]. We identified a male prevalence of 78% in bucket handle tears. Existing literature has reported male prevalence ranging from 79% to 88% [17, 18, 22].

The mean age of patients with arthroscopically proven meniscal bucket-handle tears was 35 years. In the study by Aydıngöz et al., the mean age of patients with bucket handle tears was 33, and our findings were considered relatively similar [17].

In our study, bucket handle tears were medially located in 84.31% of patients and laterally located in 15.68%. These findings are consistent with the existing literature. Wright et al. reported 82% medial (32/39) and 19.9% lateral (7/39) tears in their study [22]. Ververidis et al. reported 80.5% (29/36) medial tears and 19.4% (7/36) lateral tears, while Magee et al. reported 83.3% (25/30) medial tears and 16.6% (5/30) lateral tears [4].

Our study also revealed a similar incidence of ACL rupture associated with bucket handle tears compared to previous studies. We found a positive ACL rupture rate in 46.94% (n=23) of the patients. Wright et al. reported an incidence rate of 48% (19/39) [22]. Ververidis et al. published an incidence rate of 44% (16/36) for ACL rupture associated with bucket handle tears [18]. However, Helms et al. found a somewhat lower incidence (27%) in their research compared to similar studies [15].

In terms of MR imaging signs, in many similar studies, there are differences in the sensitivity rates of the varying signs depending on where the displaced part goes. We found the intercondylar notch sign with the highest sensitivity (84%). In the literature, its sensitivity varies from 43% to 93% [4,15,17,18,22]. We attribute this wide range mostly to the increase in image quality due to the developments in MRI technology over the years. Many studies in the literature have been done with 1.5 T MRI devices, and we considered that using a 3T device in our study may lead to different results.

In our study, perhaps the most inconsistent finding with the literature was the sensitivity of the absent bow tie sign for bucket handle tears. Other similar studies reported 88.8% to 98% sensitivities for absent bow signs [15,17,18]. But in our research, we found only 43% sensitivity of this sign. We could not find any explanation other than that the other signs on the sagittal sections might have been interpreted as absent bow tie signs and increased sensitivity.

Double PCL sign showed 56% sensitivity for diagnosing the bucket handle tears in our research. But the specificity of this sign was 100% in our study. The first describers of this sign found similar results in specificity rate [12]. And there are also similar studies with 100% specificity for double PCL signs [11,23]. At first, it was reported that the double PCL sign likely suggests the presence of medial meniscal bucket handle tears instead of lateral ones, as the ACL prevents the tear from appearing as a double PCL sign [12]. But two patients with lateral meniscus bucket handle tears were described to have the double-PCL sign; one of these patients also had an accompanying ACL rupture [17]. Both of these patients also had related contralateral meniscal bucket handle tears [17].

The ‘’V sign’’ showed a sensitivity of 72% in our study. We have not found any similar study on this sign in the literature after it was first described [5]. They suggested describing a sign that is visible at the point where the dislocated fragment (handle) forms a right angle with the intact meniscus and resembles the letter ‘’V’’ [5]. There were no signs distinctive to the axial plane of imaging that have been mentioned in the literature before. Additionally, they stated that this MRI sign had a 72% sensitivity rate just as we found.

The double ACL sign exhibited the lowest sensitivity (1.9%) for MR interpretation in our study. Another study examined five male patients who displayed a double ACL appearance on sagittal oblique MR imaging [24]. Their findings implied that detecting a double ACL sign on sagittal MR images could indirectly indicate a lateral meniscal bucket-handle rupture [24]. Two case reports in the literature also discuss this sign [20,21], supporting its role as an indicator of lateral meniscal bucket-handle tears. In our study, we identified two instances of the double ACL sign, but only one of them was confirmed as a lateral meniscal bucket-handle tear through arthroscopy. To comprehensively assess the prevalence of this sign in relation to lateral meniscal bucket-handle tears, further investigation is needed.

In our study, almost all of the MRI signs we looked at showed more than 90% specificity. And that brings us to the point that adding these markers to the radiologists' repertoire is an important detail in bucket handle tears.

We also would like to mention the limitations of our study. We have only included patients who underwent arthroscopy at the same hospital, and the patients' MRIs were retrospectively evaluated by the consensus of two radiologists. We have not assessed the intra-observer and inter-observer agreements. As a result, the findings may not be generalizable to other surgical practices or institutions. Because our study design was retrospective and because the interpreters knew that it includes only patients with arthroscopic tears, it may not have been possible to accurately prove the correlation between the MRI results and the presence or absence of bucket-handle tears.

## Conclusions

In conclusion, our study shows that MRI is a reliable imaging modality for diagnosing bucket-handle tears. With 84% sensitivity, the intercondyler notch sign showed the highest sensitivity among the eight MRI signs we studied. The high specificity (>90%) of most of the MRI findings in the study also highlights the importance of including them to the repertoire of radiologists for bucket handle tears.
